# Supplementary material for: Network Analysis of a Membrane-Enriched Brain Proteome across Stages of Alzheimer’s Disease
Source: Proteomes. 2019 Aug 27;7(3):30. doi: 10.3390/proteomes7030030 (PMC6789842; doi:10.3390/proteomes7030030)
Supplement: Supplementary file 1 [file proteomes-07-00030-s001.zip › Higginbotham_MembraneProteomeManuscript_FigureS1_FINAL_Revised.pdf]

proteomes

MDPI

Research Article

Network Analysis of a Membrane-Enriched Brain Proteome in Alzheimer's Disease

Lenora Higginbotham[1*], Eric B. Dammer[2*], Duc M. Duong[2], Erica Modeste[2], Thomas Montine[3], James J. Lah[1], Allan I. Levey[1], and Nicholas T. Seyfried[1,2,#]

[1] Department of Neurology, Emory University School of Medicine, Atlanta, GA 30322
[2] Department of Biochemistry, Emory University School of Medicine, Atlanta, GA 30322
[3] Department of Pathology, Stanford University School of Medicine, Stanford, CA 94305

* Both authors contributed equally to this manuscript.

\# Correspondence: nseyfri@emory.edu; Tel.: 1-404-712-9783

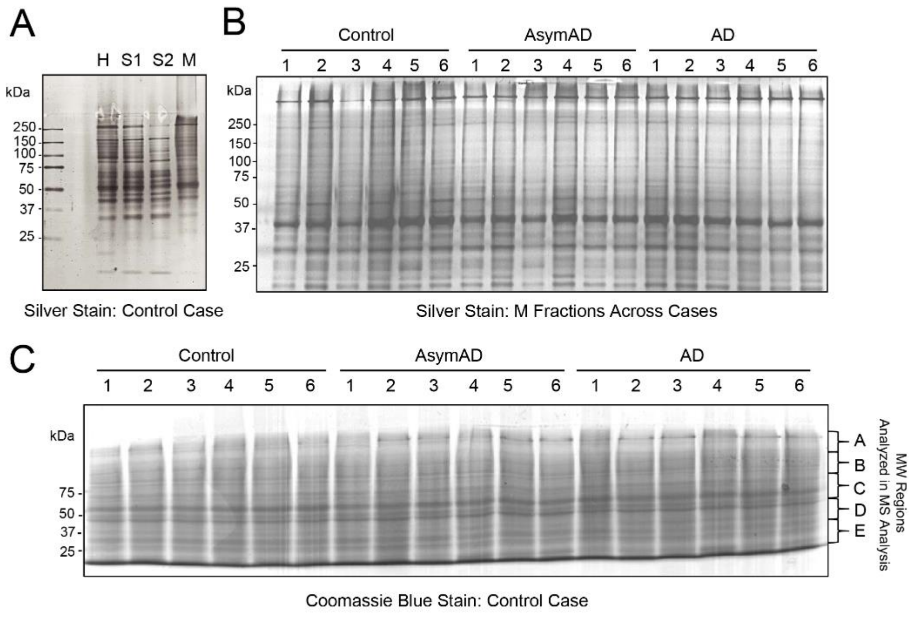

Figure S1. Preparation of Membrane-Enriched Samples. (A) Silver stain analysis of sequential fractions of an individual control case demonstrated differential banding between the cytosol-enriched S2 and membrane-enriched M fractions. (B) Silver stain analysis of the M fractions from the 18 analyzed cases demonstrated consistent protein integrity and intensity throughout. (C) Coomassie-stained SDS-page gel of the 18 alkylated M fractions. Prior to proteomic analysis, each sample lane was cut into five gel bands corresponding to the approximate molecular weight (MW) ranges shown on the right. Abbreviations: H, Homogenate; S, Supernatant; P, Pellet; MW, Molecular Weight.

© 2019 by the authors. Submitted for possible open access publication under the terms and conditions of the Creative Commons Attribution (CC BY) license (http://creativecommons.org/licenses/by/4.0/).
